# Meta-analysis of randomized controlled trials comparing single-stage laparoscopic versus two-stage endoscopic management followed by laparoscopic cholecystectomy of preoperatively diagnosed common bile duct stones

**DOI:** 10.1097/MD.0000000000041902

**Published:** 2025-03-14

**Authors:** Mohamed Ali Chaouch, Sarra Ben Jemia, Bassem Krimi, Dora Lippai, Mehdi Chahed, Amine Gouader, Faiza Khemissa

**Affiliations:** a Department of Visceral and Digestive Surgery, Monastir University Hospital, Monastir, Tunisia; b Department of Hepato-gastroenterology, Perpignan Hospital, Perpignan, France; c Department of Visceral Surgery, Perpignan Hospital, Perpignan, France.

**Keywords:** choledocholithiasis, endoscopic retrograde cholangiopancreatography, gallstones, laparoscopic cholecystectomy, laparoscopic common bile duct, stones in the common bile duct

## Abstract

**Background:**

Common bile duct stones are a significant public health issue that often requires surgical intervention. Two primary surgical techniques for addressing these conditions are laparoscopic common bile duct exploration (LCBDE) and endoscopic retrograde cholangiopancreatography (ERCP) followed by laparoscopic cholecystectomy (LC) in 1 or 2 stages, respectively. This systematic review and meta-analysis compared the efficacy and results of these 2 techniques.

**Methods:**

A systematic review and meta-analysis of randomized clinical trials followed the PRISMA and AMSTAR 2 guidelines. Literature research were performed in the Cochrane Library, PubMed/MEDLINE, Embase, and Google Scholar up to March 1, 2023.

**Results:**

Fourteen studies with a total of 1849 patients were included. The success rate was similar between LCBDE (872/919 patients) and ERCP followed by LC (866/930 patients) (odds ratio [OR] = 1.31; 95% confidence interval [CI] [0.76, 2.25], *P* = .33). There was no significant difference in residual stones (OR = 0.71; 95% CI [0.28, 1.83], *P* = .48), mortality (OR = 0.55; 95% CI [0.14, 2.14], *P* = .39), morbidity (OR = 0.87; 95% CI [0.66, 1.16], *P* = .36) or hospital stay (mean difference = -1.31; 95% CI [-2.89, 0.26], *P* = .10) between the 2 groups. Meta-analysis found no significant differences between LCBDE and ERCP followed by LC in terms of success rate, residual stones, mortality, morbidity or hospital stay.

**Conclusion:**

Both techniques are viable options for the treatment of choledocholithiasis and gallbladder stones. More multicentric randomized controlled trials are recommended to confirm these findings and explore long-term outcomes.

## 1. Introduction

Biliary stones are becoming a serious public health problem. Its incidence is about 20% in developed countries.^[1]^ In addition to the treatment of gallbladder stones, the treatment of the common bile duct stone (CBD), choledocholithiasis, is controversial despite various national guidelines.^[2–4]^ Currently, there is a significant change in the management of this disease. It depends essentially on the timing of the diagnosis: preoperatively, intraoperatively, or postoperatively.^[5,6]^ Preoperatively, the diagnosis is commonly established by imaging features. If complicated, it is associated with a high morbidity and mortality rate.^[7]^ The most appropriate treatment is chosen according to the patient’s comorbidities, symptomatic or asymptomatic conditions, and the emergent or elective diagnosis.^[8–10]^ In general, 2 different approaches are employed. A single-stage technique of Laparoscopic Common Bile Duct Exploration (LCBDE) and a two-stage technique of Endoscopic Sphincterotomy followed by Laparoscopic Cholecystectomy (ES). Both methods have benefited from new advances in laparoscopy and endoscopy, making it possible to resolve many previously complicated situations. Despite the existence of several randomized controlled trials (RCTs), there is insufficient evidence to establish the best approach. For this reason, we conducted this study to allow the highest available level of evidence in this area. This systematic review and meta-analysis aimed to compare LCBDE and ES through success rate, recurrent stone rate, mortality, morbidity, hospital stay, and conversion.

## 2. Methods

We conducted this systematic review with meta-analysis according to the 2020 PRISMA guidelines (Preferred Reporting Items for Systematic Review and Meta-analysis)^[11]^ and the AMSTAR 2 guidelines (assessing the methodological quality of systematic reviews).^[12]^ The review protocol was registered in PROSPERO before the study (CRD42023413082).

### 2.1. Electronics searches

The last electronic search of the relevant literature was conducted on March 01, 2023, with no language restriction. Trials were sought in the following databases: “Cochrane Library,” “United States National Library of Medicine,” “PubMed/MEDLINE,” “Excerpta Medica Database,” “Embase,” and “Google Scholar.” Keywords used were: “choledocholithiasis,” “common bile duct stones,” “laparoscopic exploration of common bile ducts,” “gallstones,” “laparoscopic cholecystectomy,” “endoscopic retrograde cholangiopancreatography,” ‘morbidity, “mortality,” and “residual stones.” We use the Boolean markers “and” and “or.” The reference lists of the articles obtained were checked for eligible clinical trials.

### 2.2. Study selection

*Randomized clinical* trials comparing laparoscopic exploration of the single stage CBD and cholecystectomy with two-stage retrograde endoscopic retrograde cholangiopancreatography followed by laparoscopic cholecystectomy (LC) for stones of the CBD in elective settings. Only articles published in a peer-reviewed journal were considered. Data from descriptive studies, reviews, editorial letters, case series (fewer than 10 cases), abstracts only, and comments were excluded.

### 2.3. Participants/population

Adults (aged over 18 years) of either sex treated for CBD stones with gallbladder in place.

### 2.4. Intervention group or laparoscopic group

Single stage laparoscopic exploration of the CBD and cholecystectomy.

### 2.5. Control group or endoscopic group

Two-stage retrograde endoscopic cholangiopancreatography followed by LC.

### 2.6. Outcomes

The main outcomes were the success rate and the residual stones. Secondary outcomes were mortality, morbidity, and hospital stay. Mortality and morbidity were assessed during the first 30 days of operative treatment. The remaining stones of the CBD were considered when diagnosed during the follow-up period.

### 2.7. Study selection

Two authors independently reviewed all abstracts from studies that met the inclusion criteria. Disagreements were resolved by discussion after consulting a third member of the review team.

### 2.8. Evaluation of study quality and risk of bias

The quality assessment of all studies that met the selection criteria was independently assessed by 2 authors using consolidated standards of reporting trials.^[13]^ For the risk of bias in the RCTs, we used the Cochrane tool for bias assessment to assess the risk of bias in randomized trials (RoB2).^[14]^ We evaluated bias in 5 distinct domains (randomization process, deviations from intended interventions, bias in the measurement of outcome, bias to missing outcome data, bias in selecting the reported results, and overall bias). Within each domain, 1 or more signaling questions lead to judgments of “low risk of bias,” “some concerns,” or “high risk of bias.”

### 2.9. Data extraction

Two authors independently extracted the different data from studies that met the inclusion criteria. Disagreements were resolved by discussion after consulting a third member of the review team.

### 2.10. Certainly assessment of evidence

Two authors independently assessed the evidence. We use the grading of recommendations, assessment, development, and evaluation guidelines to rate the quality of the evidence.^[15]^ We considered the limitations of the study constancy of effect, imprecision, indirectness, and publication bias. We assessed the certainty of evidence as high, moderate, low, or very low. If appropriate, we considered the following criteria to improve the certainty of evidence: large effect, dose-response gradient, and plausible confounding effect. We use the methods and recommendations described in Sections 8.5 and 8.7 and Chapters 11 and 12 of the Cochrane Handbook for systematic reviews of interventions. GRADEpro GDT software prepared the “Summary of findings tables.” We explain the reasons for downgrading or upgrading certain included studies using footnotes with comments.

### 2.11. Assessment of heterogeneity

To assess heterogeneity, 3 strategies were used:

1. The Cochrane Chi² test (Q test), Tau2, which is the variance of true effects, and 95% predictive interval (index of dispersion) to estimate the degree of heterogeneity.^[16]^ We calculated the predictive interval using a comprehensive meta-analysis prediction interval.
2. Graphical exploration with funnel plots.^[17]^
3. Sensitivity analysis with a subgroup analysis when applicable.

### 2.12. Evaluation of effect size

We used the Review Manager Web statistical package from the Cochrane Collaboration for meta-analysis.^[18]^ We selected the standard mean difference as an effective measure for continuous data. For dichotomous variables, odds ratios (OR) with 95% confidence intervals (95% CI) were calculated. A random-effects model was used. The significance threshold was fixed at 0.05. We tested the interaction between relevant factors and effect size estimates.

## 3. Results

### 3.1. Literature research

**Figure 1.**
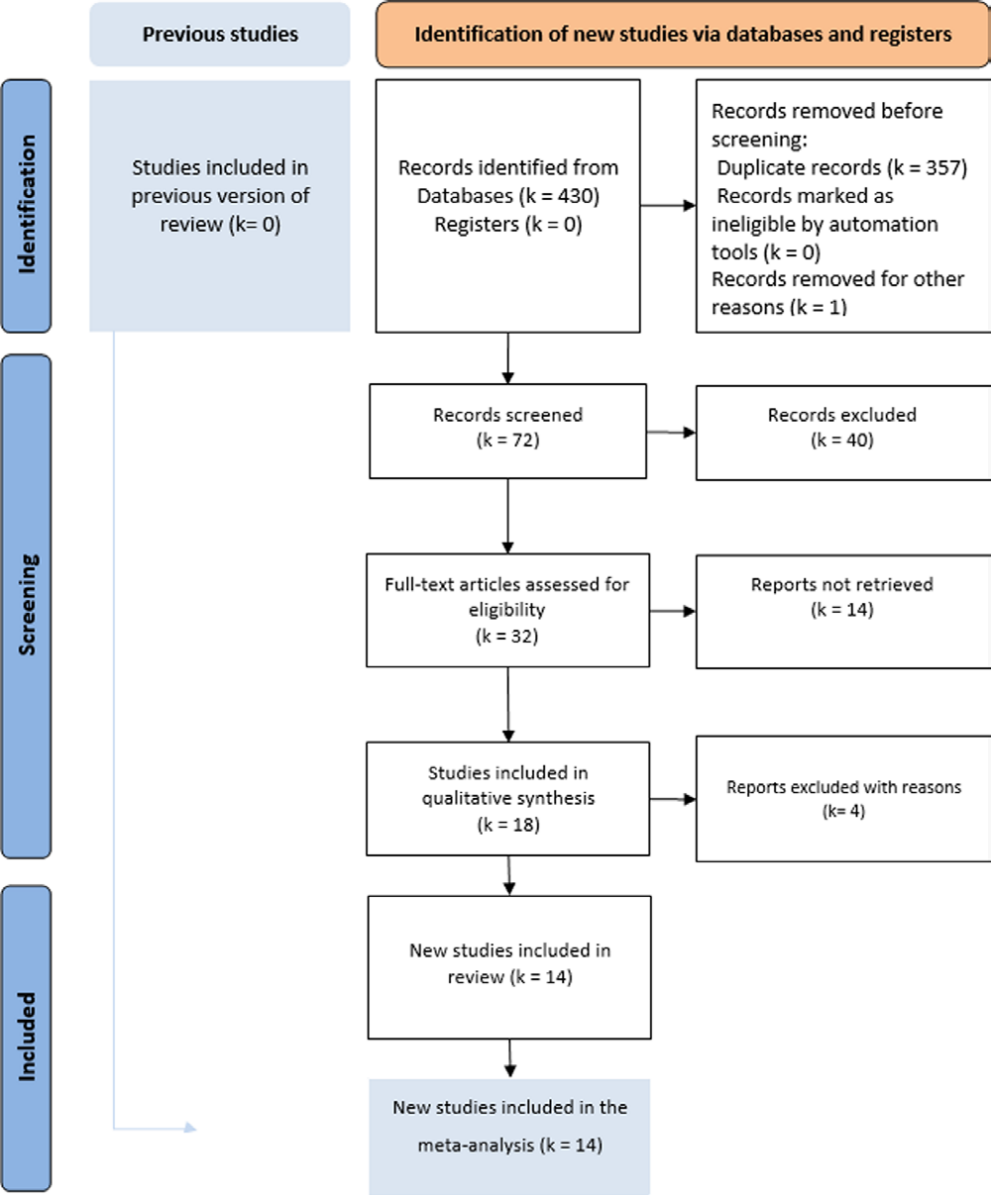
PRISMA flow diagram of bibliographic research. PRISMA = preferred reporting items for systematic review and meta-analysis.

The literature search identified 18 potentially eligible studies (Fig. 1). Fourteen studies^[19–32]^ were retained^[19–32]^ and 4 studies were excluded for the following reasons: 3 meta-analyses^[33–35]^ and 1 systematic review.^[36]^ A list of the retained studies is summarized in Table 1. These articles were published between 1999 and 2023. They included 1849 patients, 919 in the laparoscopic group, and 930 in the endoscopic group. Demographic data for the included patients are reported in Table 2. The sex ratio of the included patients ranged between 0.21 and 0.75 with a female predominance in all included studies. The mean age ranged between 39.9 and 74.3 years in the laparoscopic group and between 39 and 75.9 years in the endoscopic group. The quality assessment is also presented in Table 1. The risk of bias was individually reported in the different figures of the forest plots for each result.

**Table 1:** Demographic data from the different included randomized controlled trial.

Study	Origin	Journal	Year of publication	Study period	Sample size	LCBDE + LC	ERCP + LC	Patients with confirmed stones	Mean age (LCBDE + LC/ERCP + LC) (years)	Sex ratio (male/female)	Mean BMI (LCBDE + LC/ERCP + LC) (Kg/m²)	Study conducted by	Measured outcomes	CONSORT
Aldardeer^[19]^	Egypt	International Surgery of Journal	2019	July 2017–December 2018	150	75	75	–	–	–	–	Surgeon	Hospital stay, success rate, post-op complications, operation mean time, Stone clearance, and mortality	15/25
Bansal 2010^[20]^	India	Surgical Endoscopy	2010	July 2007–April 2008	30	15	15	15/15	47.1/39	0.33	–	Gastroenterologist	Success rate hospital stay morbidity complications	15/25
Bansal 2014^[21]^	India	Surgical Endoscopy	2014	February 2009 –October 2012	168	84	84	84/84	45.1/43	0.4	25.1/26	Gastroenterologist	Success rates mean operative time overall hospital stay number of procedures and cost of postoperative complications	17/25
Cuschieri^[23]^	European countries	Surgical Endoscopy	1999	1994–1997	300	150	150	109/99	19–88/18–89	0.39	25.7/27.3	Surgeon	Success rate post-op complications morbidity mortality hospital stay	18/25
Ding^[24]^	China	Journal of Gastrointestinal Surgery	2014	Jan 2002–Dec 2005	221	110	111	110/111	58/57	0.48	–	Surgeon	Success rate mortality morbidity post-op complications long-term follow-up outcomes	17/25
Ferulano^[32]^	Italy	Advances in Endoscopic Surgery	2011	Jan 1996-Jun 2010	124	62	62	45/39	53/55	0.21	–	Surgeon	Success rates mean operative time mortality morbidity hospital stay	16/25
Gonzalez^[37]^	Cuba	Endoscopy international open	2016	Nov 2007-Nov 2011	201	99	101	43/45	56.3/57.7	–	–	Gastroenterologist	Success rate stone clearance mean operative time post-op complications hospital stay morbidity mortality	18/25
Koc^[25]^	Turkey	The American journal of surgery	2013	Jan 2008-Sep 2010	120	60	60	60/60	51.5/54.9	0.33	24.6/25.1	Surgeon	Total operation time success rate hospital stay post-op complications stone clearance	16/25
Lv^[26]^	China	Surgical Endoscopy	2016	Feb 2014-Apr 2015	58	29	29	29/29	61.3/63.5	0.62	–	Gastroenterologist	Success rates hospital stay cost of hospitalization post-op complications	19/25
Noble^[27]^	United Kingdom	Journal of laparoendoscopic & advanced surgical techniques	2009	2000–2006	91	44	47	38/38	74.3/75.9	0.47	–	Surgeon	Hospital stay success rate post-op complications mortality	18/25
Rogers^[28]^	United States	Archives of surgery	2010	Sept 1997-Jun 2003	122	61	61	17/15	39.9/44.6	0.29	–	Surgeon	Efficacy of stone clearance, Length of procedure, morbidity, overall cost, operative time, patient acceptance, and hospital stay	16/25
Samir^[31]^	Egypt	The Egyptian Journal of Surgery	2023	Jan 2019–May 2020	107	54	53	54/53	56/51	0.75	28.5/26.8	Surgeon	Hospital stay, success rate post-op complications operation meantime	14/25
Sgourakis^[29]^	Greece	Minerva Chirurgica	2022	April 1997–August 2000	78	36	42	28/32	43–88/46–89	0.4	-		Success rate, mortality, morbidity, number of procedures, conversion rate, and hospital stay	16/25
Zou^[30]^	China	Videosurgery and Other Miniinvasive Techniques	2022	Jul 2018–Jun 2020	80	40	40	40/40	64.9/67.8	0.59	24.6/25.1	Surgeon	Hospital stay success rate post-op complications operation meantime cost of hospitalization Stone clearance	19/25

BMI = body mass index, CONSORT = consolidated standards of reporting trials, ERCP = endoscopic retrograde cholangiopancreatography, LC = laparoscopic cholecystectomy, LCBDE = laparoscopic common bile duct exploration.

**Table 2:** Summary of findings table.

Outcomes	No of participants (studies)	Certainty of the evidence (GRADE)	Relative effect (95% CI)	Anticipated absolute effects	
				Risk with endoscopy	Risk difference with laparoscopy
Mortality	1849 (14 RCTs)	⨁⨁⨁◯ Moderate*	OR 0.55 (0.14–2.14)	8 per 1000	3 fewer per 1000 (6 fewer to 8 more)
Morbidity	1825 (14 RCTs)	⨁⨁⨁◯ Moderate*	OR 0.87 (0.66–1.16)	143 per 1000	16 fewer per 1000 (44 fewer to 19 more)
Success rate	1849 (14 RCTs)	⨁⨁⨁⨁ High	OR 1.31 (0.76–2.25)	931 per 1000	15 more per 1000 (20 fewer to 37 more)
Residual stone	1063 (7 RCTs)	⨁⨁⨁◯ Moderate*	OR 0.71 (0.28–1.83)	47 per 1000	13 fewer per 1000 (33 fewer to 36 more)
Hospital stay	1179 (9 RCTs)	⨁⨁◯◯ Low*^,^†	–	The mean hospital stay was 0	MD 1.31 lower (2.89 lower to 0.26 higher)
Conversion	1559 (12 RCTs)	⨁⨁⨁◯ Moderate*	OR 0.90 (0.43–1.89)	59 per 1000	6 fewer per 1000 (33 fewer to 47 more)

*GRADE Working Group grades of evidence*

*High certainty*: we are very confident that the true effect lies close to that of the estimate of the effect. *Moderate certainty*: we are moderately confident in the effect estimate: the true effect is likely to be close to the estimate of the effect, but there is a possibility that it is substantially different. *Low certainty*: our confidence in the effect estimate is limited: the true effect may be substantially different from the estimate of the effect. *Very low certainty*: we have very little confidence in the effect estimate: the true effect is likely to be substantially different from the estimate of effect.

The risk in the intervention group (and its 95% confidence interval) is based on the assumed risk in the comparison group and the relative effect of the intervention (and its 95% CI).

CI = confidence interval, GRADE = grading of recommendations, assessment, development, and evaluation, MD = mean difference, OR = odds ratio, RCTs = randomized controlled trials.

* Small sample size.

† Existing heterogeneity among the studies.

### 3.2. Success rate

**Figure 2.**
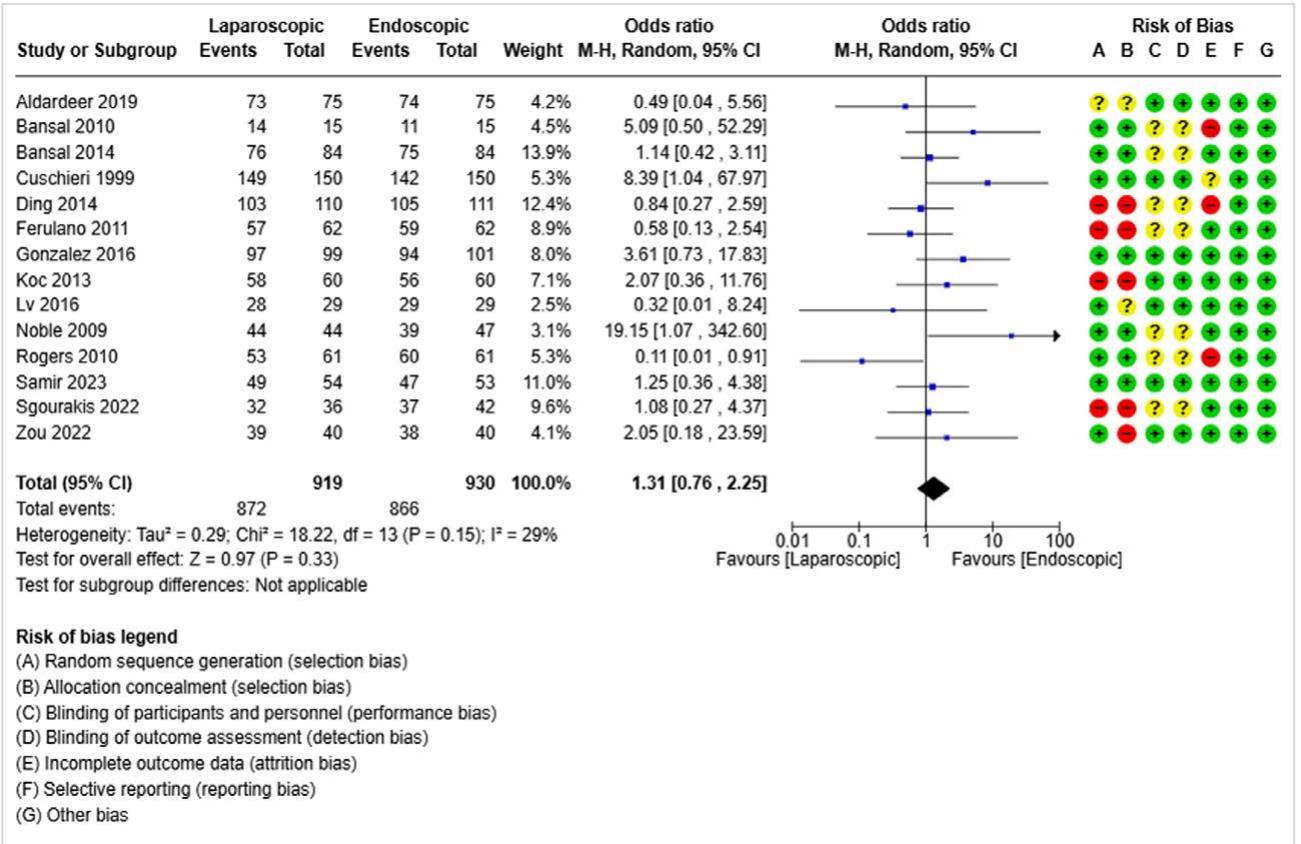
Forest plot of success rate.

All retained studies reported a success rate.^[19–32]^ It was reported in 872 out of 919 patients in the laparoscopic group and 866 out of 930 patients in the endoscopic group. The pooled analysis did not show differences between the 2 groups in terms of the success rate (OR = 1.31; 95% CI [0.76, 2.25], *P* = .33) (Fig. 2). There was a low heterogeneity rate among the different studies (Tau²=0.29).

### 3.3. Residual stones

**Figure 3.**
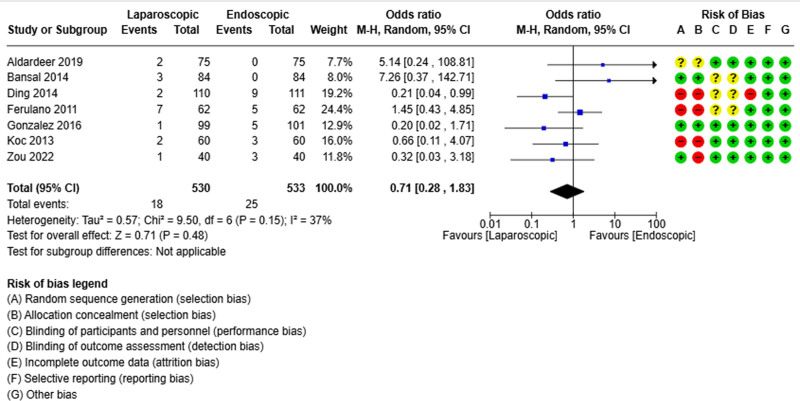
Forest plot of residual stone.

Seven studies reported the residual stone rate.^[19,21,22,24,25,30,32]^ It was reported in 18 of 530 patients in the laparoscopic group and 25 of 533 patients in the endoscopic group. The pooled analysis did not show differences between the 2 groups in terms of the residual stone rate (OR = 0.71; 95% CI [0.28, 1.83], *P* = .48) (Fig. 3). There was a low heterogeneity rate among the different studies (Tau²=0.57).

### 3.4. Mortality

**Figure 4.**
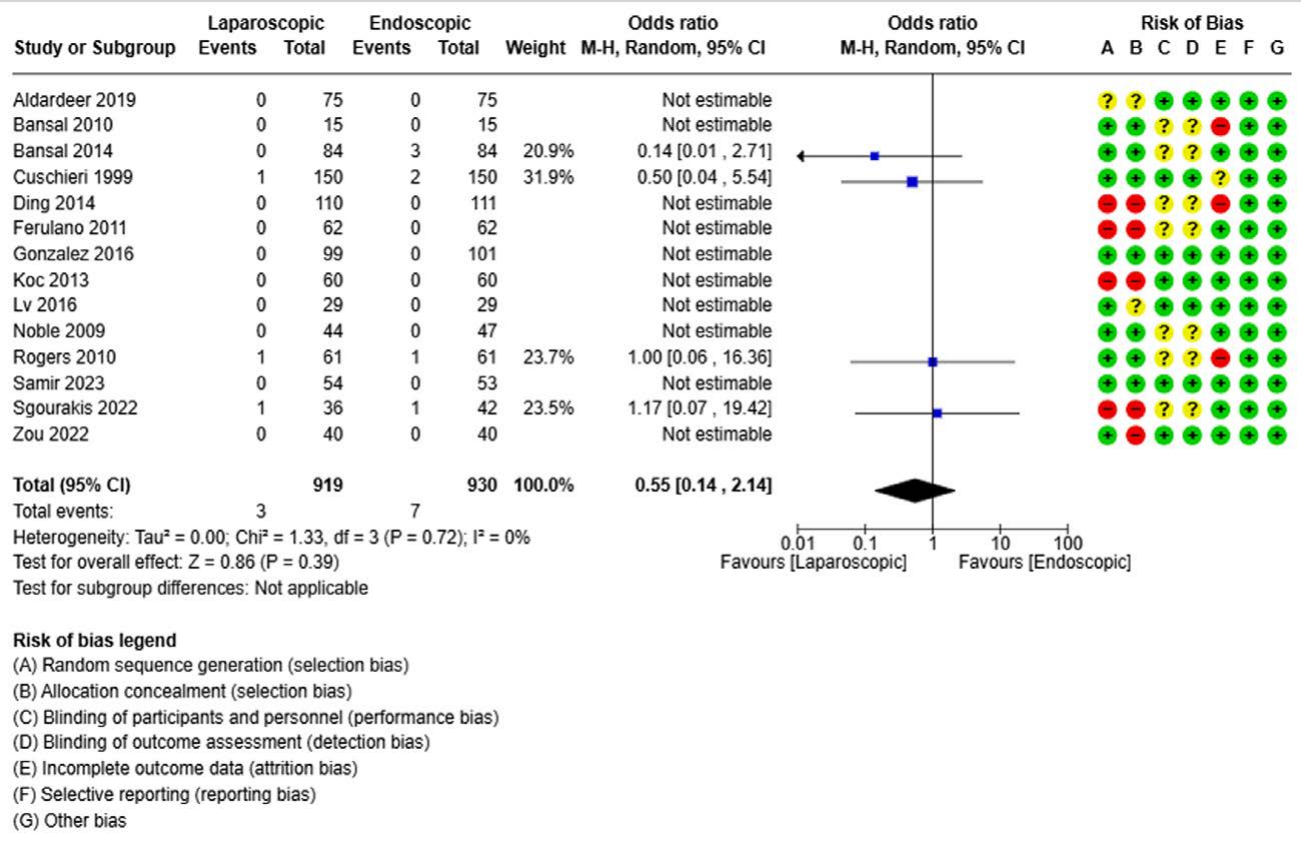
Forest plot of mortality rate.

All retained studies reported the mortality rate.^[19–32]^ It was reported in 3 of 919 patients in the laparoscopic group and 7 of 930 patients in the endoscopic group. The pooled analysis did not show differences between the 2 groups in terms of mortality (OR = 0.55; 95% CI [0.14, 2.14], *P* = .39) (Fig. 4).

### 3.5. Morbidity

**Figure 5.**
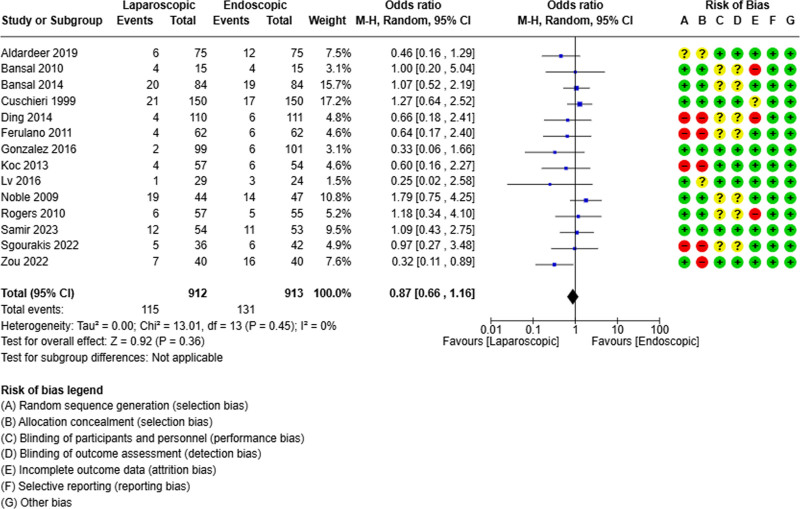
Forest plot of morbidity rate.

All retained studies reported the morbidity rate.^[19–32]^ It was reported in 115 out of 912 patients in the laparoscopic group and 131 out of 913 patients in the endoscopic group. The pooled analysis did not show differences between the 2 groups in terms of morbidity (OR = 0.87; 95% CI [0.66, 1.16], *P* = .36) (Fig. 5).

### 3.6. Hospital stay

**Figure 6.**
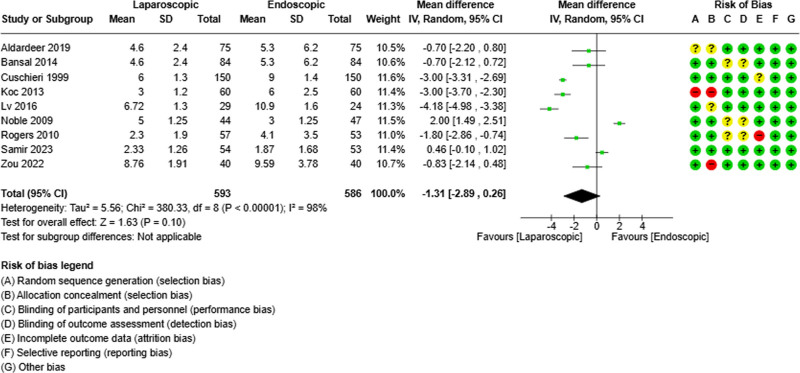
Forest plot of hospital stay.

Nine studies evaluated the length of hospital stay.^[19,21,23,25–28,30,31]^ They compared 593 and 586 patients in the 2 groups. There were no significant differences between the 2 groups in terms of hospital stay (mean difference = -1.31; 95% CI [-2.89, 0.26], *P* = .01). There was low heterogeneity between the different studies (Tau²=5.56) (Fig. 6).

### 3.7. Conversion

**Figure 7.**
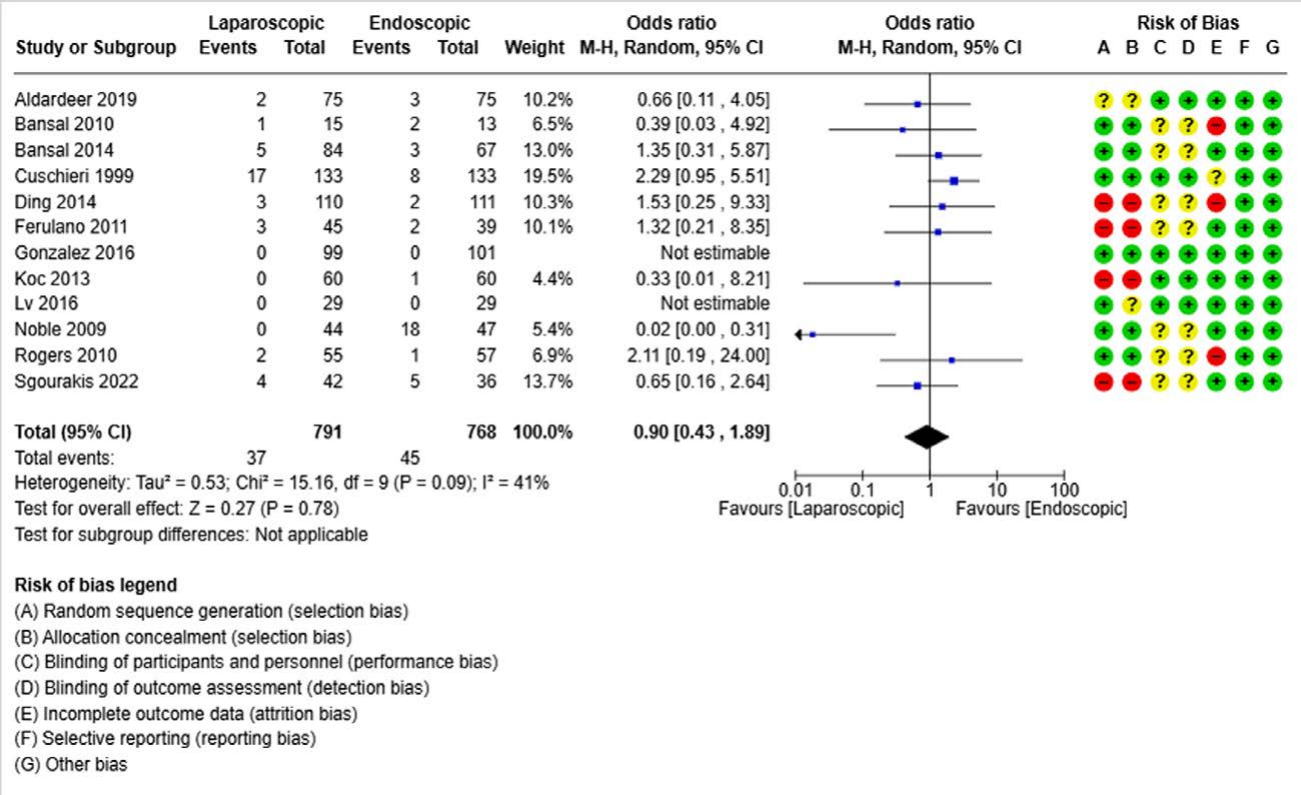
Forest plot of conversion.

Twelve studies reported the conversion rate.^[19–21,23–29,32,37]^ It was reported in 37 of 791 patients in the laparoscopic group and 45 of 768 patients in the endoscopic group. The pooled analysis did not show differences between the 2 groups in terms of conversion (OR = 0.90; 95% CI [0.43, 1.89], *P* = .78). There was low heterogeneity between the different studies (Tau²=0.53) (Fig. 7).

Quality assessment of the included studies and reporting of the effects of laparoscopic single stage CBD exploration and cholecystectomy for CBD stone compared to laparoendoscopic two-stage endoscopic CBD stone extraction followed by cholecystectomy.

The quality assessment is presented in Table 1. The risk of bias was reported in the different forest plots individually for each outcome. The summary of the findings table is presented in Table 2. This systematic review and meta-analysis concluded that when single-stage laparoscopic exploration of the CBD and cholecystectomy for the CBD was compared to laparoendoscopic two-stage endoscopic extraction of stone from the CBD followed by cholecystectomy, there were no differences between the 2 groups in terms of success rate, rate of recurrent stone rate, mortality, morbidity, hospital stay and conversion.

## 4. Discussion

CBD stones affect 10% up to 15% of patients with gallstone disease.^[1]^ Mainly 2 strategies were used. Although each of the 2 offers distinct advantages, our meta-analysis, which included 14 RCTs with a total of 1849 patients, revealed a notable similarity between the 2 methods. Both techniques show the same success rates, without significant differences in terms of residual stones, mortality, morbidity, or length of hospital stay.

The success rate was 94.8% for the LCBDE group and 93.1% for the ES group. The odds ratio found in our study suggests that the slight numerical difference in favor of LCBDE is not statistically significant. These results have demonstrated that both procedures are highly effective. This finding expertise also highlighted the worldwide endoscopist and surgeon and the possibility to allow patient’s choice of procedure and patient selection, which are important factors in achieving individualized treatment plans. An additional important outcome in evaluating the long-term success of procedures is the recurrent stone rate. In this meta-analysis, we found a low stone recurrence rate in all included studies, with moderate differences between the 2 groups (3.4% vs 4.7%). This insignificant difference could be attributed to a more comprehensive examination of the bile duct. The cohesion of these findings in multiple studies reinforces the validity of the data, and the low heterogeneity (Tau² = 0.57) could be explained by the absence of standardized diagnostic imaging tools or the use of ultrasound endoscopy in all studies. Bansal et al^[21]^ and Koc et al^[25]^ attributed some advantages to the LCBDE. It can reduce the chance of leaving residual stones by direct view and clear stones in a single procedure, especially in the case of complex biliary anatomy or multiple stones. We should mention that in some difficult cases a conversion is considered. It is a decisive element in evaluating the achievability of laparoscopic LCBDE cholecystectomy after endoscopic retrograde cholangiopancreatography (ERCP) to treat choledocholithiasis. In this meta-analysis, the pooled analysis showed similar conversion rates. The low heterogeneity observed in the studies could be explained by some factors such as stone size, complexity of the biliary anatomy and patient-specific risk factors instead of the choice of the technique itself. Conversion in LCBDE is often allied to technical difficulties in stone extraction or inadequate visualization and surgeon expertise, oppositely conversion in the ES group can occur due to failed endoscopic access, impacted stones, difficult biliary anatomy or in the case of pancreatitis or cholangitis.

Regarding mortality, our study found that its rate was low for both groups (0.3% for LCBDE vs 0.8% for ERCP + LC). These results confirm that, in terms of mortality, the choice between LCBDE and ERCP + LC should be based more on patient characteristics and surgeon expertise than on safety concerns. Koc et al^[25]^ found a higher mortality rate in the ES group (0.7% vs 0.3%), this difference was attributed to the associated risks of performing 2 separate procedures and to some additional specific complications of the ES. Then another important outcome was to assess morbidity. In this meta-analysis, the pooled data concluded the absence of a difference between the 2 groups. However, the distinct procedural approaches of the 2 techniques can contribute to different types of complications and morbidities. Although LCBDE allows direct visualization and removal of stones, it is not without specific risks. The main morbidities associated with LCBDE include bile leaks, which occur when there is an inadvertent injury to the bile duct during exploration and additional surgical site infections. Bansal et al^[21]^ and Koc et al^[25]^ reported that such complications were approximately rare but associated with a longer operative time. In addition, laparoscopy requires pneumoperitoneum, which has additional specific complications. On the other hand, in the case of a two-stage procedure, the morbidity caused by ES mainly involves essentially pancreatitis estimated between 3 and 5%,^[22,26]^ cholangitis, and perforations. These findings were of great concern in the case of elderly patients with various comorbidities. The risk of postoperative complications is greater, and we should try to apply complete and rapid treatment with a shorter operative time and a shorter hospital stay. LCBDE in the study by the elderly was evaluated in the Parra-Membrives et al.^[38]^ This study compared the postoperative outcomes of LCBDE in the elderly versus the general population and there were no differences between the 2 groups. Gantois et al^[39]^ compared the one-stage LCBDE with the two-stage procedure in patients 75 and more. There was a similar mortality, morbidity and conversion rate with better efficacy in terms of clearing the CBD of lithiasis and requiring fewer anesthetic procedures in LCBDE. Furthermore, CBD with packed stones appears to be a risk factor for failure. For these reasons, it may be more suitable to suggest a one-stage LCBDE in the case of elderly patients, especially in CBD packed with multiple stones that could require in the majority cases a biliary and digestive anastomosis.

Finally, these 2 procedures have common safety profiles, and their similar morbidity rates reflect the high standard of care provided in the centers that perform these procedures.^[24]^ Although there are procedure-specific complications, such as bile leaks that are more common with LCBDE and pancreatitis that are more common with ERCP, most of these complications are low and manageable with appropriate perioperative care and surgical expertise. This strength of morbidity in both techniques depends mainly on factors like patient selection, the complexity of the biliary anatomy, the presence of multiple stones, and the level of experience of the surgical team. Then, the choice between these techniques should not be made based on presumed differences in morbidity alone, even though the bottom line suggests that both groups are associated with certain procedure-specific risks. Instead, patient risk factors, anatomical considerations, and available surgical expertise should be individually considered to minimize the likelihood of complications and optimize patient outcomes.

In this meta-analysis, the hospital stay for patients with LCBDE was shorter with 1.31 days without significant statistical difference. Although LCBDE is a single-stage procedure and ERCP followed by LC involves 2 stages, the pooled results showed that hospital stay is comparable. Primarily, the impact of postoperative complications and the need for patient monitoring rather than the number of procedural steps underline that the duration of hospitalization is more often controlled by factors such as postoperative complications, patient comorbidities, the application of enhanced recovery protocols and the management of complex cases more than the choice of technique.^[20,25,26,36]^

The results of our meta-analysis align with previous studies comparing single-stage LCBDE with two-stage ERCP followed by LC. Li et al^[40]^ conducted a meta-analysis of RCTs and reported no significant differences in stone clearance, postoperative morbidity, mortality, or hospital stay between the 2 techniques.^[40]^ Similarly, Singh et al^[33]^ found that LCBDE was associated with a shorter hospital stay and lower rates of technical failure compared to ERCP followed by LC, though mortality and morbidity rates were comparable.^[33]^ Our study differs from these meta-analyses in several ways. Firstly, we included more recent studies, expanding the evidence base to account for advancements in surgical techniques and perioperative management. Secondly, while Li et al^[40]^ and Singh et al^[33]^ emphasized procedural success and failure rates, our analysis also considered the impact of recurrent stones and the conversion rate between procedures. Despite slight variations in methodology and patient selection, our findings reinforce the notion that both techniques are equally effective, with procedural choice depending on surgeon expertise, institutional resources, and patient-specific factors. Moreover, our study expands on the role of LCBDE in high-risk patients. While Singh et al^[33]^ recommended LCBDE primarily for “good-risk” patients where expertise is available, our findings suggest that LCBDE can be safely applied to a broader population when performed by experienced surgeons. Future RCTs should explore the long-term outcomes and cost-effectiveness of these approaches to further refine clinical guidelines.

This meta-analysis presents a robust comparison between single-stage LCBDE and the two-stage approach involving ERCP followed by LC. Its strengths include a rigorous methodology that adheres to the PRISMA and AMSTAR 2 guidelines, a comprehensive literature search in multiple databases, and the inclusion of 14 RCTs with 1849 patients, ensuring a high level of evidence. Additionally, the study provides a balanced assessment of key outcomes such as success rate, residual stones, mortality, morbidity, and hospital stay, demonstrating that both techniques are viable treatment options. However, some limitations should be acknowledged. The study lacks long-term follow-up data, making it difficult to conclude recurrence and late complications. Furthermore, while included studies were assessed for bias, heterogeneity in patient selection, procedural expertise, and definitions of key outcomes may introduce variability. Lastly, the absence of standardized imaging tools and differences in surgical and endoscopic expertise between centers may have influenced the results. Future large-scale, multicenter trials with longer follow-ups are needed to further refine clinical guidelines.

## 5. Conclusions

These results suggest that the choice of method emphasizes more on the patient’s specific conditions, such as his available resources and the surgeon’s expertise than on the inherent superiority of one method over the other. However, more large-scale multicenter RCTs are needed to confirm these findings and better understand long-term results, to refine clinical guidelines.

## Author contributions

**Conceptualization:** Mohamed Ali Chaouch, Sarra Ben Jemia, Dora Lippai, Mehdi Chahed.

**Data curation:** Mohamed Ali Chaouch, Sarra Ben Jemia, Dora Lippai, Mehdi Chahed.

**Formal analysis:** Mohamed Ali Chaouch, Sarra Ben Jemia, Bassem Krimi, Dora Lippai.

**Investigation:** Sarra Ben Jemia, Dora Lippai, Amine Gouader.

**Methodology:** Mohamed Ali Chaouch, Amine Gouader.

**Project administration:** Sarra Ben Jemia, Bassem Krimi.

**Resources:** Mohamed Ali Chaouch, Mehdi Chahed, Amine Gouader.

**Software:** Sarra Ben Jemia, Amine Gouader.

**Supervision:** Mohamed Ali Chaouch, Bassem Krimi, Dora Lippai, Amine Gouader, Faiza Khemissa.

**Validation:** Mohamed Ali Chaouch, Sarra Ben Jemia, Bassem Krimi, Dora Lippai, Faiza Khemissa.

**Writing – original draft:** Mohamed Ali Chaouch, Sarra Ben Jemia, Dora Lippai.

**Writing – review & editing:** Mohamed Ali Chaouch, Faiza Khemissa.
